# Supplementary material for: Industry 4.0 readiness and strategic plan failures in SMEs: A comprehensive analysis
Source: PLoS One. 2025 May 20;20(5):e0324052. doi: 10.1371/journal.pone.0324052 (PMC12091723; doi:10.1371/journal.pone.0324052)
Supplement: Attachment — Submitted filename: Rebuttal Letter for Manuscript PONE.pdf [file pone.0324052.s002.pdf]

Rebuttal Letter for Manuscript PONE-D-24-42283: "Industry 4.0 Readiness and Strategic Plan Failures in SMEs: A Comprehensive Analysis"

Dear Dr Syed Hamid Hussain Madni and Reviewers,

We want to extend our sincere gratitude for your thorough review and constructive feedback on our manuscript. We have carefully considered each comment and incorporated revisions to improve the paper's clarity, organisation, and depth. Below, we address each reviewer's comments in detail.

1. Reviewer #1

Abstract

1. **Comment**: The abstract needs a clear introduction and problem statement. Please revise it to follow a structured format, including Background, Problem, Objective, Methods, and Results.
   - **Response**: Thank you for this recommendation. We have revised the abstract to include distinct sections for Background, Problem Statement, Objective, Methods, Results, and Conclusion to improve its clarity and alignment with PLOS ONE standards.
   - **Changes Made**: As suggested, the abstract now follows a structured format (see page 1).
2. **Comment**: Please rewrite the keywords, ensuring the first word is capitalised and consider incorporating "Malaysia" or "Malaysian SMEs" for region-specific research.
   - **Response**: We have revised the keywords to enhance searchability and included "Malaysian SMEs" to emphasise regional focus.
   - **Changes Made**: Keywords updated to "Industry 4.0, Malaysian SMEs, Digital Transformation, Workforce Competency, Technology Adoption, NIMP 2030."

Introduction

1. **Comment**: The author should elaborate on the relationship between NIMP 2030 and the Industry4WRD program earlier in this section.
    - **Response**: We expanded the introduction to clarify the relationship between NIMP 2030 and the Industry4WRD program, emphasising how these initiatives collectively support SME readiness.
    - **Changes Made**: The introduction briefly discusses NIMP 2030 and Industry4WRD (see pages 2-3).
2. **Comment**: Include recent statistics on the contribution of SMEs to Malaysia's GDP and employment.
    - **Response**: We updated the introduction with recent data to reflect SMEs' role in Malaysia's economy.
    - **Changes Made**: Recent statistics on SME contribution included (see page 2).
3. **Comment**: Add the study's contribution, research questions, and an overview of the paper's organisation.
    - **Response**: We clarified the study's contribution and primary research question and added an outline of the paper's organisation at the end of the introduction.
    - **Changes Made**: Added a clear statement of contribution, research question, and paper organisation (see pages 2–3).

Literature Review

1. **Comment**: Organize the literature review into subsections (e.g., Industry 4.0 Concepts, SME Challenges, and Readiness Assessment Frameworks).
    - **Response**: We organised the literature review into thematic subsections to improve readability and focus.

- **Changes Made**: The literature review is divided into Industry 4.0 Concepts, SME Challenges, and Readiness Assessment Frameworks (see pages 3–5).

2. **Comment**: Include more recent references, particularly from 2024, to reflect the latest developments.
    - **Response**: We have added recent references from 2024 to incorporate the latest research on Industry 4.0 and SME challenges.
    - **Changes Made**: Updated references throughout the literature review (see pages 3–5).
3. **Comment**: Outline the research gap in the related works section, comparing it with existing studies.
    - **Response**: We expanded the literature review to identify the research gap and provide a critical analysis, using the referenced manuscripts as guidance.
    - **Changes Made**: The research gap is discussed in detail, highlighting how our study addresses the limitations of prior studies (see page 5).

Methodology

1. **Comment**: Provide more details on the sampling strategy, scale rationale, statistical tests, and potential limitations.
    - **Response**: We clarified the sampling strategy, rationale behind the 0-4 scale, and statistical tests and addressed data collection limitations.
    - **Changes Made**: Methodology expanded with sampling strategy, scale rationale, specific tests, and limitations (see pages 5–6).

Results

1. **Comment**: Add context for interpreting readiness scores, confidence intervals or p-values, and explain sectoral differences.

- **Response**: We added interpretive context for readiness scores and statistical significance indicators and discussed why sectors like Chemicals exhibit higher readiness.
- **Changes Made**: The results section was enhanced with additional context, confidence intervals, and sectoral analysis (see pages 6–11).

Conclusion

1. **Comment**: Discuss broader implications for developing economies and suggest areas for future research.
    - **Response**: We expanded the conclusion to discuss implications for developing economies and propose specific directions for future research.
    - **Changes Made**: Broader implications and future research directions are now included (see page 15).

General Comments

1. **Comment**: Improve grammatical and typographical accuracy, and consider professional proofreading.
    - **Response**: We have thoroughly proofread the manuscript and considered professional services to ensure language clarity and accuracy.
    - **Changes Made**: Minor grammatical and typographical errors were corrected throughout.

2. Reviewer #2

Abstract

1. **Comment**: The abstract needs to adequately demonstrate the background, purpose, methodology, and contribution.
   - **Response**: Per your guidance, we restructured the abstract to communicate these elements clearly.
   - **Changes Made**: The revised abstract will include sections for background, purpose, methodology, and contribution (see page 1).

Introduction

1. **Comment**: The introduction needs a more explicit statement of the need for this research, the contribution of the paper, and an outline of the organisation.
   - **Response**: We have revised the introduction to emphasise the need for research on Industry 4.0 readiness among Malaysian SMEs, included the study's unique contribution, and added an outline of the paper.
   - **Changes Made**: The introduction was expanded to address the need, contribution, and structure (see pages 1–2).

Literature Review

1. **Comment**: The literature review should better discuss current literature, analyse critically, and show the study's unique perspective.
   - **Response**: We organised the literature review to include current studies, provide critical analysis, and demonstrate the study's unique angle.
   - **Changes Made**: Literature review restructured and expanded with critical analysis of existing literature (see pages 2–6).

Methodology

1. **Comment**: The methodology section needs to be completed, and the methodology needs to be discussed adequately.
   - **Response**: We clarified the sampling strategy, readiness assessment scale, and specific statistical analyses performed to ensure a complete methodology.
   - **Changes Made**: The methodology expanded to provide comprehensive details (see pages 6–7).

Results and Discussion

1. **Comment**: Results and discussion sections need to be more detailed.
   - **Response**: We added interpretive context to the results and expanded the discussion to highlight broader implications, especially for other developing economies.
   - **Changes Made**: Results and discussion sections enriched with context and broader implications (see pages 7-16).

We are grateful for both reviewers' insightful comments, which have helped improve the rigour and clarity of our manuscript. We trust these revisions meet PLOS ONE's standards and welcome further feedback.

Sincerely,

Dr. Muhammad Saqib Iqbal
Assistant Professor
NUST Business School, NUST
Corresponding Author
