# Supplementary material for: Industry 4.0 readiness and strategic plan failures in SMEs: A comprehensive analysis
Source: PLoS One. 2025 May 20;20(5):e0324052. doi: 10.1371/journal.pone.0324052 (PMC12091723; doi:10.1371/journal.pone.0324052)
Supplement: Attachment — Submitted filename: Rebuttal Letter for Manuscript Feb25.pdf [file pone.0324052.s003.pdf]

Response to Reviewer Comments

Dear Dr Annamalah and Reviewer 2,

We thank you for the thoughtful and constructive feedback on our manuscript, "Industry 4.0 Readiness and Strategic Plan Failures in SMEs: A Comprehensive Analysis." We have carefully considered all comments and made revisions to improve the paper's clarity, rigour, and overall quality. Below, we detail our responses to each of the reviewer's comments:

Reviewer Comment 1:

"Introduction section fails to show the need for this research work. The same text is repeated in the methodology section and introduction section."

Response:
We have restructured the Introduction to articulate the research gap and the necessity for assessing Industry 4.0 readiness among Malaysian SMEs. In the revised Introduction, we emphasise the unique challenges SMEs face and clearly state the contribution of our study. Additionally, we have removed any redundant methodological details previously appearing in the Introduction, ensuring that the Methodology section now exclusively covers the research design and data collection procedures.

Reviewer Comment 2:

"The manuscript must describe a technically sound scientific research piece with data supporting the conclusions. Experiments must have been conducted rigorously, with appropriate controls, replication, and sample sizes. The conclusions must be drawn appropriately based on the data presented."

Response:
We affirm that the manuscript is technically sound and the data robustly supports our conclusions. Our study employed a rigorous experimental design with a sample of 506 SMEs, appropriate controls, and replication. To address this comment explicitly, we have added a new subheading in the Methodology section titled **"Technical Rigor and Data Integrity."** In this subsection, we detail our survey instrument's pilot testing and expert validation and the multiple statistical analyses (including correlation, regression, and ANOVA) performed to validate the relationships among key dimensions. These procedures ensure that our conclusions are drawn appropriately based on the data presented.

Reviewer Comment 3

"Literature review should discuss the current literature and critically analyse the literature."

Response:
We have expanded the Literature Review section to include a more comprehensive discussion of recent studies. The revised literature review now critically analyses current findings, identifies existing gaps, and positions our research as a necessary contribution to the field of Industry 4.0 readiness. This expanded discussion clarifies how our multidimensional assessment addresses limitations in prior research and enhances our understanding of the challenges and opportunities for Malaysian SMEs.

Reviewer Comment 4:

"The methodology section is incomplete. It does not discuss the method. We need to describe the factors, what items are included in data collection, etc."

Response:
We have extensively revised the Methodology section to include a detailed description of our data collection process and the measurement of key dimensions. Specifically, a new subheading, **"Data Collection and Survey Instrument,"** has been added. In this section, we describe the structured questionnaire designed to measure five key dimensions of Industry 4.0 readiness: leadership, governance, digital infrastructure, workforce competency, and strategic alignment. We explain that each item was rated on a standardised 0–4 Likert scale and provide examples of operationalising these constructs. This detailed description enhances the transparency and reproducibility of our study.

Additional Note:

The revisions made during the review process aim to clarify our methodology and emphasise the technical rigour of our research. These changes do not affect the underlying data, key findings, or the study's conclusions. Consequently, no modifications have been made to the abstract or findings.

We believe these revisions have significantly improved the clarity and quality of our manuscript. We thank you again for your valuable feedback and look forward to your favourable consideration of our revised submission.

Sincerely,

Muhammad Saqib Iqbal, PhD
Zulhasni Abdul Rahim, PhD
and Umawathy Techanamurthy, PhD
